# A dependent Bayesian Dirichlet process model for source apportionment of particle number size distribution

**DOI:** 10.1002/env.2763

**Published:** 2022-09-22

**Authors:** Oliver Baerenbold, Melanie Meis, Israel Martínez‐Hernández, Carolina Euán, Wesley S. Burr, Anja Tremper, Gary Fuller, Monica Pirani, Marta Blangiardo

**Affiliations:** ^1^ Department of Epidemiology and Biostatistics, MRC Centre for Environment and Health Imperial College London UK; ^2^ Department of Atmospheric and Oceanic Sciences Consejo Nacional de Investigaciones Cientinficas y Tecnologicas (CONICET), Centro del Mar y la Atmósfera y los Océanos (CIMA‐UBA‐CONICET), Universidad de Buenos Aires Buenos Aires Argentina; ^3^ Department of Mathematics and Statistics Lancaster University Lancaster UK; ^4^ Department of Mathematics Trent University Peterborough Ontario Canada

**Keywords:** Bayesian modeling, dependent Dirichlet process, particle concentrations, source apportionment

## Abstract

The relationship between particle exposure and health risks has been well established in recent years. Particulate matter (PM) is made up of different components coming from several sources, which might have different level of toxicity. Hence, identifying these sources is an important task in order to implement effective policies to improve air quality and population health. The problem of identifying sources of particulate pollution has already been studied in the literature. However, current methods require an a priori specification of the number of sources and do not include information on covariates in the source allocations. Here, we propose a novel Bayesian nonparametric approach to overcome these limitations. In particular, we model source contribution using a Dirichlet process as a prior for source profiles, which allows us to estimate the number of components that contribute to particle concentration rather than fixing this number beforehand. To better characterize them we also include meteorological variables (wind speed and direction) as covariates within the allocation process via a flexible Gaussian kernel. We apply the model to apportion particle number size distribution measured near London Gatwick Airport (UK) in 2019. When analyzing this data, we are able to identify the most common PM sources, as well as new sources that have not been identified with the commonly used methods.

## 1 INTRODUCTION

Air pollution is a complex mixture of solid particles, liquid droplets and gases and represents one of the most studied environmental stressors on human health. In particular, there is an extensive evidence of the negative health effects of particulate matter (PM) (Khomenko et al., 2021; Manisalidis et al., 2020). Most epidemiological studies and policy reports have focused on total PM concentration (see for instance e.g., Pope 3rd, 2000; World Health Organization, 2013). However, recent evidence has shown that mixture of particles from different sources can have different toxicity and health effects (e.g., Dai et al., 2014; Park et al., 2014; Pirani et al., 2015; Samoli et al., 2016).

Compositional data with information on the different chemical components within PM concentration is vital to disentangle the total PM into sources. However, this is expensive and not widely available worldwide monitoring site networks. As an alternative, measurement of particle number concentration (PNC) and related particle number size distribution (PNSD) have additionally received much attention and have been recently considered as a way to investigate PM sources (Hopke et al., 2022). Typically, this involves considering a wide range of particle sizes, ranging from ∼10 to 2500 nm diameter, spanning across both the ultrafine (<100 nm diameter) and fine (100–2500 nm diameter) particle ranges. Similar results have been reported when comparing sources from compositional data and PNSD, particularly for long range transport sources (Gu et al., 2011). Beddows et al. (2015) saw that compositional data and PNSD are complementary when looking at sources. A recent study looked at PNSD around Gatwick Airport (London, England), and was able to estimate direct airport sources, related to aircraft departure and landing, as well as indirect sources, for instance due to the traffic in the area (Tremper et al., 2022). Another study used PNSD to assess air pollution sources around four European cities and was able to highlight some common sources across the cities, as well as specific ones (Rivas et al., 2020). For example, sources related to fresh traffic and urban air pollution were identified in each of the four cities in analysis, while a biogenic source was only apparent in Helsinki.

Working with PNSD means dealing with a large number of correlated variables; practically, the range of sizes is split into a large number of bins and the number of particles in each bin is calculated. As there is typically correlation across the different bins, the main statistical challenge lays in reducing the high dimensional correlated data into an interpretable smaller number of sources, something which is known in the exposure science literature as *source apportionment* (SA), (Belis et al., 2014; Hopke, 2016; Viana et al., 2008). SA aims at deriving information about ambient air pollution sources based on data registered at monitoring sites, therefore allowing the quantification of individual source contributions to the pollution concentrations in the air (Krall & Chang, 2019).

### 1.1 Preliminaries on SA

Traditionally, methods for the SA problem have been dominated by two approaches (as discussed by Viana et al., 2008): source‐oriented deterministic models and receptor models. The former relies on the knowledge of emissions and physical and chemical processes of dispersion to predict air quality through the specification of deterministic models; the latter is based on statistical procedures for identifying and quantifying the sources of pollutants on the basis of mixture of chemicals measured at receptor sites. A review on SA methods by Hopke (2016), however, underlined that the deterministic models fail to provide accurate representations of the variability of chemical species and concentrations observed in the atmosphere. Commonly, receptor models for SA decompose ambient concentrations of pollutants into components based on how they co‐vary, then associating the components with different source types. Within this framework, there are a variety of approaches, which according to Schauer et al. (2006) can be positioned along the continuum of *a priori* knowledge: from chemical mass balance (CMB) at one end (Clements et al., 2016; Watson et al., 1990), if sources are known and detailed information on source profiles are available; to multivariate methods such as positive matrix factorization (PMF) (Norris et al., 2014; Paatero & Tapper, 1994) at the other, if little or nothing is known about the nature of the pollution sources.

PMF has been the most popular of a variety of multivariate methods due to the Environmental Protection Agency's historic development of PMF3.0 and PMF5.0 software (Norris et al., 2014), allowing easy user access to powerful features, including quantification of the contribution to specific sources through decomposition of the matrix of samples into factor contribution and profile matrices, (e.g., Gu et al., 2011; Hopke et al., 2022; Rivas et al., 2020; Tremper et al., 2022). PMF is a technique developed to identify common factors in a multivariate setting where non‐negative constraints are required (Paatero & Tapper, 1994). In the context of PNSD, as presented in Rivas et al. (2020), let $Y_{P\times T}$ be observed data with P size categories and T time points. Then, the PMF method assumes that

(1) $$y_{p,t}=\overset{K}{\underset{k=1}{\sum }}\lambda_{p,k}f_{k,t}+\varepsilon_{p,t},$$

with K the number of independent sources; $\lambda_{p,k}$ the particle number concentration of the pth category bin emitted by source k; $f_{k,t}$ the contribution of the kth source at time t; and $\varepsilon_{p,t}$ the residuals.

In matrix notation, model (1) can be written as $Y_{P\times T}=\Lambda_{P\times K}F_{K\times T}+\varepsilon_{P\times T}$. Notice that the observed data matrix $Y_{P\times T}$ has non‐negative entries (one cannot observe negative particulate concentrations); additionally, as we interpret the matrix $\Lambda_{P\times K}$ as source profile densities, it also has to have non‐negative entries and each column should sum to 1. Similarly, $F_{K\times T}$ is interpreted as a contribution from the k source to the total particle concentration. Eigen analysis is a common method to identify factors (e.g., principle components analysis as a particularly famous example); however, such methods do not guarantee *positive* values. The PMF method uses a penalized least‐squares approach to identify the model components, constraining both $F_{K\times T}$ and $\Lambda_{P\times K}$ to be non negative.

In practice, there are no objective criteria to select the number of factors when applying the PMF. Thus, for the different number of factors, the user compares the output, $\Lambda_{P\times K}$ and $F_{K\times T}$, using some empirical rules such as those discussed in (Tremper et al., 2022): (1) obtaining scaled residuals in the range of (−3,3); (2) checking that the resulting $F_{K\times T}$ time series show low correlation; and, most importantly, (3) assessing that $\Lambda_{P\times K}$ are physically interpretable profiles. The relative subjectivity in this approach is a limitation. Additionally, there is not a principled way of accounting for the uncertainty in the source allocation, and the method requires that the dataset is complete, hence missing data need to be removed or imputed. Finally, similar to most dimension reduction techniques, PMF relies on the assumption that the source contributions or pollution mixtures are independent over time. However, this may not be appropriate and temporal dependence can exist. This dependence could be (partially or completely) explained by covariates, particularly related to meteorology, as there is abundant evidence of the relationship between meteorological variables and the quantity of PM present in the air. For example, Borge et al. (2016) exposes a strong correlation between certain meteorological variables, such as wind and local emissions, in a campaign study from a hotspot in Madrid. A similar study was done on different locations in Buenos Aires (Pineda Rojas et al., 2020) where the authors found a significant correlation between PM${}_{10}$ and meteorological variables like lower sky cover and relative humidity. Furthermore, the authors showed that wind speed was the variable explaining most of the air pollution variation across sites.

### 1.2 Bayesian approaches to SA and novelty

Within a Bayesian perspective, a number of contributions have been proposed in the context of factor analysis similar to PMF, mainly developed to apportion PM compositional data (e.g., Hackstadt & Peng, 2014; Heaton & Scott, 2010; Lingwall et al., 2008; Park et al., 2001; Park & Oh, 2015; Tang et al., 2020), along with specific computational packages (e.g., Park et al., 2021). For example, Park et al. (2001) included in the model an autoregressive component to account for temporal correlation in the data, while Park and Oh (2015) explicitly accounted for nonnegativity constraints on the source contributions and source compositions, in both parameter and model uncertainty estimation. Lingwall et al. (2008) proposed a generalized Dirichlet distribution as a flexible prior for the source composition profiles, while Heaton and Scott (2010) described a generalized Dirichlet distribution in order to model time‐varying source contributions. Nikolov et al. (2011) proposed a multiplicative factor analysis with a latent mixed model structure on the latent source contributions, including meteorological covariates and temporal correlation for a fixed number of sources. Hackstadt and Peng (2014) proposed a multivariate receptor model, incorporating information on source emission compositions and contributions from known sources. Recently, Tang et al. (2020) developed a hierarchical mixed effect model, assuming the number of sources to be known, based on informative prior distributions of latent variables that summarize the knowledge of experts and previous studies.

Here we propose a modeling approach to apportion PNSD and allow for a data driven selection of the number of sources, while keeping the option of including covariates on the latent source contributions. It is based on the Dirichlet process (DP; (Ferguson, 1973)) prior, characterized by having Dirichlet distributed finite dimensional marginal distributions. This prior is particularly useful as it can be used to model the uncertainty about the functional form of the distribution for a model parameters (Görür & Edward Rasmussen, 2010). Because the distributions drawn from a DP are discrete, but cannot be described using a finite number of parameters (Teh, 2010), it allows us to identify latent sources, without need to specify their number *a priori*. In this study, we extend the DP framework by allowing dependence from meteorological conditions, specifically wind speed and wind direction, in the source allocation while smoothing the process using a flexible Gaussian kernel (Dunson & Park, 2008). Finally, adopting a Bayesian perspective, our model can naturally accommodate missing data in the number of particle sizes, which are predicted by the model. We demonstrate the effectiveness of our proposed approach by apportioning time‐series of PNSD data gathered near London Gatwick Airport (UK) in 2019.

The structure of the article is as follows. In Section 2, we start by presenting the data used to illustrate the method. Then, in Section 3 we present our modeling approach and in Section 4 we present the results. Finally in Section 5 we provide a discussion and future directions.

## 2 DATA DESCRIPTION AND PRE‐PROCESSING

Hourly measurements of particle concentrations for a wide range of sizes were obtained from a monitoring station (Lat: 51.166, Long: −0.168) close to London Gatwick Airport, which is around 40 km south of London. The station is positioned within a residential area approximately 1.5 km from the runway at Gatwick. The airport is considered as one of the most important emission sources in the region, largely due to the considerable amount of aircraft traffic. A measuring campaign was run between January 25, 2019 and September 11, 2019, with the particle size distribution measured in 107 different size bins ranging from 14.6 to 661.2 nm. Details about the measurement campaign and the instrumentation used can be found in (Tremper et al., 2022). Hourly wind speed and wind direction for London Gatwick Airport were imported from the National Oceanic and Atmospheric Administration's Integrated Surface Database through the worldmet package (Carslaw, 2022) for the R programming environment (R Core Team, 2021). Time‐series concentrations of the following pollutants were also measured at the same monitoring site: nitric oxides (total oxides of nitrogen (NO${}_{X}$), nitrogen dioxide (NO${}_{2}$), and nitrogen oxide (NO)), particulate matter (total particulate matter <10μm (PM${}_{10}$), base fraction of PM (PMFB), and volatile fraction of PM (PMFR)), carbon (black carbon measured in infrared transmission (CBLK), black carbon measured in ultra‐violet transmission (CBUV), and carbon from wood burning (CWOD)).

**FIGURE 1:**
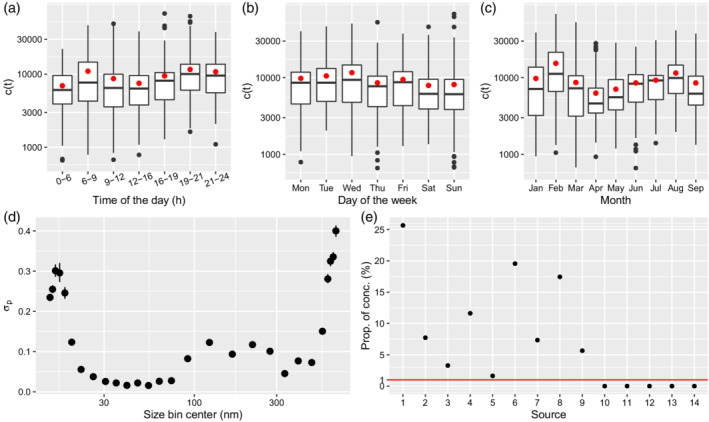
Model results: Boxplot and mean (red dot) of total particle concentration by time of the day (a), day of the week (b), and month (c); measurement error $\sigma_{p}$ for each size bin (d), and the proportion of total concentration in each source with a limit at 1% (e).

We reduced the number of size bins for computational feasibility. Consecutive bins were aggregated as follows. For the pth bin we computed the correlation between $y_{p,t}$ and $y_{p+q,t}$, for q=1,2,…; when this correlation was below a certain threshold, τ, we summed the concentrations and considered this as a new bin where the value represents the total particle concentration across both size bins (see correlation matrix in Supplementary material, Section 1, Figure 1).

Setting a threshold of τ=0.97 leads to 28 distinct size bins with a larger number of bins being aggregated in the center of the distribution. For example, while the smallest size bin at 14.6 nm remains unchanged, nine size bins from 105.5 to 140.7 nm were combined. Dimension reduction across time was performed similarly. For this we denote $y_{p,t}$ as $y_{p,\text{day},\text{hour}}$ to emphasize how t is split into days and hours. Consecutive hours were aggregated as follows. We computed the correlation between $y_{p,\text{day},\text{hour}}$ and $y_{p,\text{day},\text{hour}+h}$, for h=1,2,…,24—hour and for each p, using data across all days. Then for a specific p we averaged the concentration across the hours if the corresponding correlation was above 0.97 (see Supplementary Material, Section 1, Figure 1).

A reduction to 7 daily time steps is sufficient to represent the typically two distinct daily peaks in particle concentrations, going from 0 to 6, 6 to 9, 9 to 12, 12 to 16, 16 to 19, 19 to 21, and 21 to 24 h. Groups vary in duration from 2 h (19 to 21) to 6 h (0 to 6). In total the data reduction results in the change from 5494 time steps × 107 (587,858) size bins to 1604 time steps × 28 size bins (44,912), a data driven data reduction of 92.4%.

Zero concentration measurements were recorded in 921 cases. These can be considered as artifacts of concentrations below the detection threshold of the instruments as the particle concentration is strictly positive. These were replaced with half of the minimum non‐zero observed value in the corresponding size bin. Missing data in particle concentration are related to downtime of the measuring device and therefore occur for all size bins simultaneously, with overall 7.6% of observations being missing. The missingness occurred in five separate events ranging in duration from 1 to 8 days and these events were left missing to be predicted by the model. There was no missing data for the nitric oxides, on the order of 1% missing for CBLK, CBUV, CWOD, PMFB, and PMFR, and 10% missing for PMFR. Missing wind direction and speed data were imputed with the respective mean value at 8 time steps for wind direction and at 1 time step for wind speed. The number of missing wind observations is too small (less than 1% for both variables) for a model of missingness and mean imputation was chosen to keep a continuous time‐series and avoid discarding any data. A sensitivity analysis to the imputation of wind observations was performed by discarding the time steps with missing wind data, with no change observed.

## 3 BAYESIAN SOURCE APPORTIONMENT MODEL

We propose a SA model for the particle size concentration distribution, allowing for an unknown number of sources and including covariates in the source allocation process. For the pth bin (p=1,…,28) and tth time point (t=1,…,1604), we model the particle concentration $y_{p,t}$ as follows:

(2) $$\begin{array}{ll} \log(y_{p,t}) & ∼𝒩\left(\log(\mu_{p,t}),\sigma_{p}\right), \end{array}$$

(3) $$\begin{array}{ll} \mu_{p,t} & =\underset{k}{\sum }\lambda_{p,k}f_{k,t}, \end{array}$$

where $\sigma_{p}$ represents the measurement error, which we assume size‐specific. Note that, due to the log‐transformation in (2), our formulation implies a multiplicative error structure, differently from (1), which assumes additive errors. The $\lambda_{p,k}$ describes the source profile density and analogously to the PMF, it gives the proportion of particles from source k in size bin p.

We specify a finite approximation of a DP (Ferguson, 1973) that avoids an *a priori* selection of the number of sources, but encourages the accumulation of contributions in fewer of them. To that end, we replace the source contribution $f_{k,t}$ as $f_{k,t}=s_{k,t}c_{t}$, where $s_{k,t}$ is the proportion of the total particle concentration at time t contributed by source k, and $c_{t}$ is the total particle concentration at time t. We model the parameter $c_{t}$ as being normally distributed on the log‐scale with a common mean and standard deviation $\log(c_{t})∼𝒩(\mu_{c},\sigma_{c})$.

### 3.1 Dirichlet prior for source contribution

We take a non‐parametric approach by assuming a dependent DP (DDP) as a prior for $s_{k,t}$. The DDP is a flexible class of covariate‐dependent random probability distributions (Dunson & Park, 2008; MacEachern, 1999; Quintana et al., 2022). In the following, we first briefly review a constructive representation of the DP known as the stick‐breaking representation, then we turn to the description of our proposed approach, which generalizes the stick‐breaking process by replacing the Beta random variables with a more complex form. Generally speaking, realizations from a DP are random infinite discrete probability distributions (Ferguson, 1973). Let G denote a random probability measure, $G_{0}$ be a continuous distribution function and α be a positive real number, that is, $\alpha \in {\mathbb{R}}_{0}^{+}$. Then $G|\alpha ,G_{0}∼\text{DP}(\alpha ,G_{0})$ is a random probability measure with the same support as $G_{0}$, the base measure. $G_{0}$ is the expected value of the process and α is the precision (or concentration) parameter, which is interpreted as an inverse variance (i.e., smaller values for α lead to sparser distributions). We consider a stick‐breaking representation of the DP, provided by Sethuraman (1994), and defined as follows:

(4) $$G=\overset{\infty }{\underset{k=1}{\sum }}s_{k}\delta_{\theta_{k}},$$

where $\delta_{\theta_{k}}$ is the Dirac measure (point mass) at $\theta_{k}$ and $\theta_{k}\;\overset{iid}{˜}\;G_{0}$. The probability mixing weights are defined as $s_{1}=\xi_{1}$ and $s_{k}=\xi_{k}\underset{l<k}{\prod }(1-\xi_{l})$ for k>1, with $\xi_{k}\;\overset{iid}{˜}\;\text{Beta}(1,\alpha )$. Here $\left\{s_{k}\right\}$ and $\left\{\theta_{k}\right\}$ are independent. With the representation (4), one can verify that $G∼\text{DP}(\alpha ,G_{0})$. The name of this DP representation derives from an analogy given by breaking off pieces of length $s_{k}$ from a stick of unit length, where the breakpoints ($\xi_{1},\xi_{2},\ldots \;$) are randomly sampled from a Beta distribution. The mixture probabilities break the stick into a potentially infinite number of pieces, such that they sum to the unity.

The mixing weights can be allowed to vary across time by drawing the probabilities from an identical stick‐breaking DP at each time step t, that is, $\xi_{k,t}\;\overset{iid}{˜}\;\text{Beta}(1,\alpha )$. This captures time variations in the breakpoints corresponding to time variations in source contributions.

In this article, we exploit the temporal information contained in the data by allowing the mixing weights, $s_{k,t}$, to be a function of covariates (Dunson & Park, 2008; Quintana et al., 2022).

We consider wind speed and direction as our main covariates since meteorological factors heavily affect the PNSD. For computational feasibility, we implement the model with finite approximation to the infinite stick‐breaking process (Ishwaran & Zarepour, 2000), that is k=1,2,…,K. The covariates influence only the first K−1 mixing weights as the Kth is set to take on the remaining probability up to 1. Therefore, the structure in (4) is modified as follows:

(5) $$\begin{array}{ll} G_{t} & =\overset{K}{\underset{k=1}{\sum }}s_{k,t}\delta_{\theta_{k}},\\ s_{1,t} & =\xi_{1,t},\\ s_{k,t} & =\xi_{k,t}\underset{l<k}{\prod }(1-\xi_{l,t}),\;k=2,\ldots ,K,\\ \xi_{k,t} & =w_{k,t}\cdot \eta_{k,t},\;k=1,\ldots ,K-1,\\ \xi_{K,t} & =1,\\ \eta_{k,t} & ∼\text{Beta}(1,\alpha ),\;k=1,\ldots ,K-1,\\ \theta_{k} & \;\overset{iid}{˜}\;G_{0}, \end{array}$$

where $w_{k,t}$ is a kernel that incorporates weights dependent on wind speed and wind direction. When $w_{k,t}=1$ the model becomes a standard time varying DP without time dependency. The kernel has to be constructed flexibly enough to capture the expected dependency of the mixing weights on the covariates while preventing overfitting. The mode of the kernel distribution needs to be 1 to maintain the validity of the stick‐breaking process. We expect source contributions to increase with a specific wind direction that is reflective of the location of the source and a specific wind speed that reflects the distance. In detail, we introduce a kernel that is Gaussian in wind speed and in a sinus transformation of the wind direction to allow for the dependence of a source on wind that follows the criteria above. Given mode and bandwidth parameter in wind speed and wind direction, we use this kernel to incorporate wind speed in m/s ($\left\{ws_{t}\right\}$) and wind direction in degrees clockwise from north ($\left\{wd_{t}\right\}$) both aggregated at the same 7 daily time steps as the observed data. Explicitly,

(6) $$\begin{array}{ll} w_{k,t}=\exp\left(\frac{{\left(\nu_{1,k}-{\text{ws}}_{t}\right)}^{2}}{2\cdot h_{1,k}}\right)\cdot \exp\left(\frac{\sin{\left((\nu_{2,k}-{\text{wd}}_{t})\cdot \pi /360\right)}^{2}}{2\cdot h_{2,k}}\right), & \end{array}$$

where $\nu_{1,k}$ and $\nu_{2,k}$ are the kernel modes (knots) for the two wind‐related covariates, and $h_{1,k}$, $h_{2,k}$ the corresponding bandwidths, which control the spread for source k both in angular and speed direction. By contrast to PMF and to the Bayesian model formulation in (4), K does not refer to the actual number of sources but to the maximum number of sources. The actual number of sources is not a parameter of the model but determined using the empirical rule that a source needs to have a contribution $\sum_{t}f_{k,t}$ larger than a minimum threshold.

### 3.2 Prior specification

Model formulation was completed by putting minimally informative priors on all hyper‐parameters. This ensures that the inference is driven by the data. The mean and variances of the log transformed total concentration parameter $c_{t}$ are modeled as $\mu_{c}∼𝒩(0,\sigma =10)$ and $\sigma_{c}∼\Gamma (1,0.001)$ respectively; the measurement error variance are modeled as $\sigma_{p}^{2}∼\Gamma (1,1)$.

The source profiles were modeled using Jeffrey's Dirichlet prior, $\lambda_{k}∼D(0.5),k=1,\ldots ,K$. The α parameter can be understood as an inverse variance and broadly speaking, it controls the number of components. Being a key parameter in the model, we first consider a minimally‐informative Gamma prior (α∼Γ(1,1)), but perform sensitivity analysis by changing it to both more (α∼Γ(1,10)) and less informative priors (α∼Γ(1,0.1)), which would give higher and lower probability to a smaller number of components (Supplementary material, Section 2.1).

Vague Gamma distributions were assumed on the kernel bandwidths $h_{1,k}^{-1}∼\Gamma (1,0.001)$, and $h_{2,k}^{-1}∼\Gamma (1,0.001)$. Finally, uniform priors on the kernel modes $\nu_{1,k}∼U(0,\max(\text{ws}))$, and $\nu_{2,k}∼U(0,360)$ were assumed, with upper limits that ensure the values can span across the entire empirical range of the covariates.

### 3.3 Implementation

The model was implemented in the nimble (de Valpine et al., 2017) probabilistic programming language, and run under R 4.1.2 (R Core Team, 2021). All code is available on GitHub (Baerenbold et al., 2022).

Running our model is computationally demanding, but still feasible on a standard personal computer, requiring less than 6 GB of RAM with a runtime in the order of four days on an Intel Core i7‐8565U processor at 4.6 GHz peak frequency for 60,000 steps. To achieve reasonable runtimes we performed dimension reduction in the number of size bins and daily time steps as a pre‐processing step. While aggregating size bins appears less consequential due to the very high temporal correlation of at least 0.97 between size bins that were combined, there is considerable variation between the hours aggregated within a single time step. Running the model without the time aggregation step increases the required RAM to 18 GB and would take an estimated 14 days to run making it still feasible on a modern workstation, while a model without size bin aggregation requires in excess of 60 GB of RAM and in an optimistic linear extrapolation of time required per size bin gives a run‐time of more than 50 days. Note that the same number of cores were made available to the Nimble sampler in all conditions, (4 cores/8 threads). The memory usage seems to be associated with the category size increase, forcing more variables and more complex environment for the sampler to work in.

We ran two separate inferences, one with 120,000 iterations and 60,000 burn‐in with 1 in 60 thinning, and one with 60,000 iterations and 30,000 burn‐in with 1 in 30 thinning, and the outputs are available on Zenodo (Baerenbold & Burr, 2022). Inferences were compared by whether they identified both the same sources and the same total number of sources (Supplementary material, Section 2.2). Model convergence was assessed in each chain separately using visual inspection of trace plots and Gelman–Rubin diagnostics after splitting the chain in half (Supplementary material, Section 3).

## 4 RESULTS

Comparing the posterior distributions of the identified source profiles using the two runs we found that they were consistent (Supplementary material, Section 2.2, Figures 8 and 9). Additionally, changing the prior specification on the α parameter gave similar results (Supplementary material, Section 2.1, Figure 7). Here, we present the data analysis findings based on the longer run.

### 4.1 Overview

We first present the results related to the non‐source specific parameters. Focusing on the total particle concentration c, the posterior distribution of the total particle concentration (estimated by the $c_{t}$ parameter in the model) presents temporal variation, which is showed in Figure 1a–c for time of the day, day of the week and month. The concentration is highest for typical commuting hours during the day (6–8 and 19–20), reflecting potential traffic sources, as well as for weekdays, particularly Monday to Wednesday (Figure 1a,b). The pattern over months is less clear, with the highest values in February and August, potentially reflecting different sources (Figure 1c). The posterior mean of the size‐specific standard deviation parameters $\sigma_{p}$ show larger uncertainty at the extremes, below 20 nm and above 500 nm sizes (Figure 1d). This is expected as below 20 nm and above 200 nm there is more uncertainty due to the measurement process (Wiedensohler et al., 2012). The relatively low uncertainty between 200 and 500 nm is likely related to the aggregation of a larger number of size bins in this range.

We compare predicted values based on the model with the observed data for small particles of 14 nm, mid‐sized particles around 100 nm, and large particles of 661 nm (Supplementary material, Section 1, Figures 4–6). The predictions match the data well and in agreement with Figure 1d the increased measurement error in larger size bins is clearly visible in the more extreme values that are not captured by the prediction curve. Imputation of missing data is informed by the wind kernel but not by other temporal patterns in the source contributions.

Moving to the sources estimates, the model finds 9 non‐empty sources (see Figure 1e) ranging from just above 1% to more than 25% of the total concentration.

**FIGURE 2:**
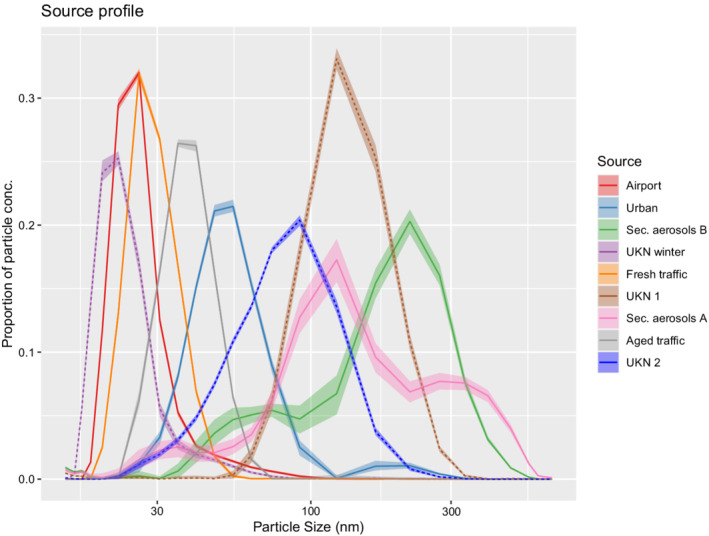
Particle size distribution for the 9 sources identified by the model. Solid lines represent the sources which were also found using PMF in Tremper et al. (2022), while dashed lines are new findings.

**FIGURE 3:**
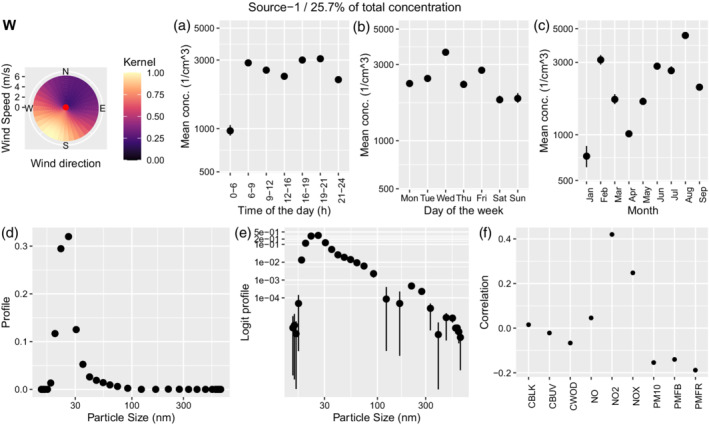
Characterization of source 1, which was identified as airport. We show the corresponding wind kernel (W), mean variation across time of the day (a), day of the week (b), month (c), profile on natural scale (d), profile on the logit scale (e), and correlation with externally measured air pollutants (f).

The correlation matrix between the sources over time shows coefficients ranging from −0.22 to 0.67 (Supplementary material, Section 1, Figure 3). Notable positive correlations above 0.5 are between sources with numbers 1 and 6; 2 and 7; 3, 9, and 5; and 9 and 8. Positive correlations are to a degree expected due to common meteorological causes while stronger negative correlations would indicate source splitting. Figure 2 shows the source profiles, by means of the proportion of particle concentration that each sources contributes to over the range of particle sizes. The profiles cover the entire range of sizes and all 9 sources are clearly distinct. Corresponding wind kernels suggest that the wind plays a role in the source attribution (Supplementary material, Section 1, Figure 2). Note that due to finite approximation of the DPP, the model estimates only wind kernels for the first K−1 sources.

### 4.2 Sources previously identified by PMF

In an earlier sampling campaign conducted on the same site between July and October 2018, six distinct sources were identified using PMF, namely, airport, fresh traffic, aged traffic, urban, secondary aerosol A, and secondary aerosol B (Tremper et al., 2022). Of our nine identified sources, six closely match the characterizations found by PMF.

#### Airport related source

Source 1 is the largest, covering 25.7% of the total concentration compared with 17.1% for the airport factor based on PMF, although the analyses are for different time periods. Figure 3, panel W shows that contributions from this source are strongest when wind blows from the south‐west, which is the source direction of London Gatwick Airport. There are clear temporal patterns: particle concentration is 3 times lower during the night in accordance with approximate flight activity at Gatwick Airport and there is an indication, albeit weak, of morning and evening peaks (Figure 3a). The source contribution is slightly higher during weekdays than on weekends with two peaks on Wednesday and Friday (Figure 3b). Over the year we can see variations by a factor of about 3 which can be related to both seasonal wind patterns and variations in source output (Figure 3c). The main peak of the distribution is between 20 and 30 nm, with potentially an additional peak in the 200 to 300 nm range visible in the logit transformed profile (Figure 3d,e). The source is moderately positively correlated with source 6, weakly positively correlated with NO${}_{2}$, and NO${}_{X}$, and weakly negatively correlated with PM${}_{10}$, PMFB, and PMFR.

#### Fresh and aged traffic

**FIGURE 4:**
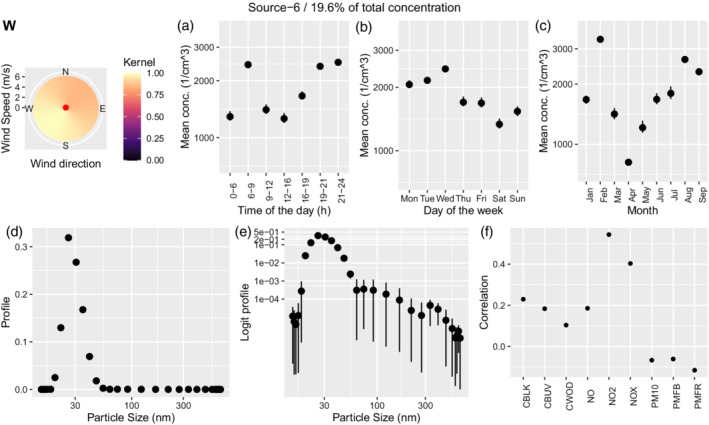
Characterization of source 6 which was identified as fresh traffic. We show the corresponding wind kernel (W), mean variation across time of the day (a), weekday (b), month (c), profile (d), profile on the logit scale (e), and correlation of the time‐series of concentration with externally measured air pollutants (f).

**FIGURE 5:**
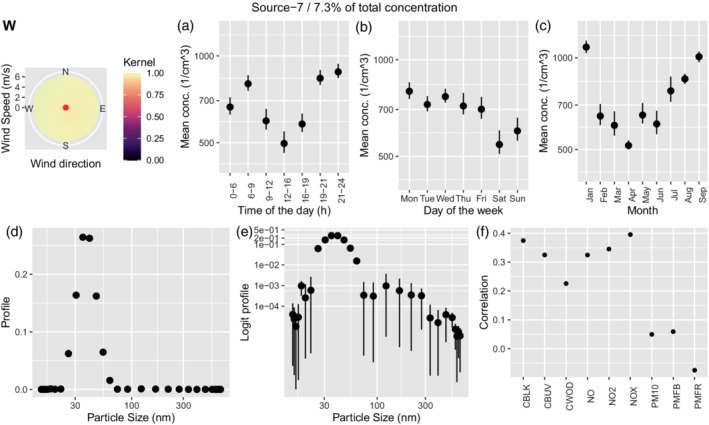
Characterization of source 7 which was identified as aged traffic. We show the corresponding wind kernel (W), mean variation across time of the day (a), weekday (b), month (c), profile (d), profile on the logit scale (e), and correlation of the time‐series of concentration with externally measured air pollutants (f).

Traffic is represented in two sources: source 6 represents freshly emitted traffic particles, while source 7 identifies traffic particles after coagulation (aged traffic). Both sources share a similar daily pattern, with two clear peaks and lower values during the middle of the day and the night (Figures 4 and 5a). Additionally, they both show the highest values on Monday–Wednesday, then reducing on Thursday–Friday and reaching the lowest values during the weekend (Figures 4 and 5b). However, while fresh traffic contributes for 19.6% of the total concentration, aged traffic covers only 7.3%. The size distribution is centered around slightly lower values for fresh traffic (between 20 and 30 nm), while it reaches 35 nm for aged traffic. Finally, while fresh traffic shows a weak relationship with wind from south‐west, aged traffic does not seem to be related to wind coming from a specific direction (Figures 4, 5, panel W). A moderate correlation between the airport source and the fresh traffic source has been reported previously by Tremper et al. (2022). Consequently, source 6 shows correlations to other pollutants similar to source 1.

#### Urban

**FIGURE 6:**
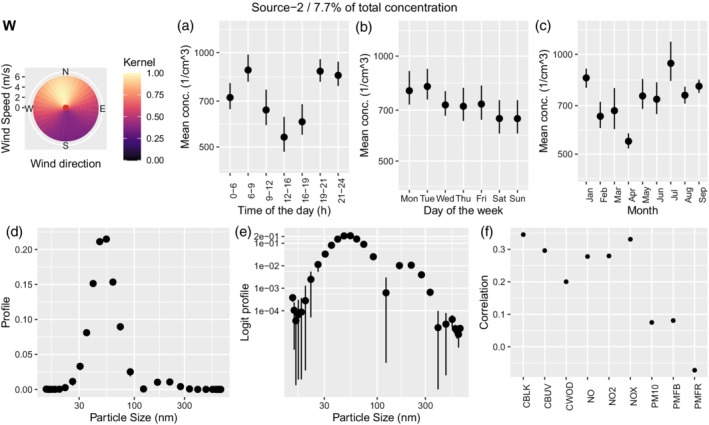
Characterization of source 2 which was identified as urban. We show the corresponding wind kernel (W), mean variation across time of the day (a), weekday (b), month (c), profile (d), profile on the logit scale (e), and correlation of the time‐series of concentration with externally measured air pollutants (f).

Source 2 contributes 7.7% of total concentration and its characteristics are presented in Figure 6. It shows a relationship with wind coming from north, which is where London is located, and a somewhat bimodal regime, with a higher peak at around 50 nm, but also a lower peak at around 200 nm. The concentration decreases only slightly during the night and shows the lowest value in the middle of the day. It has moderate correlation with aged traffic and similarly to that source, it shows correlation to black carbon and nitric oxides.

#### Secondary aerosols

**FIGURE 7:**
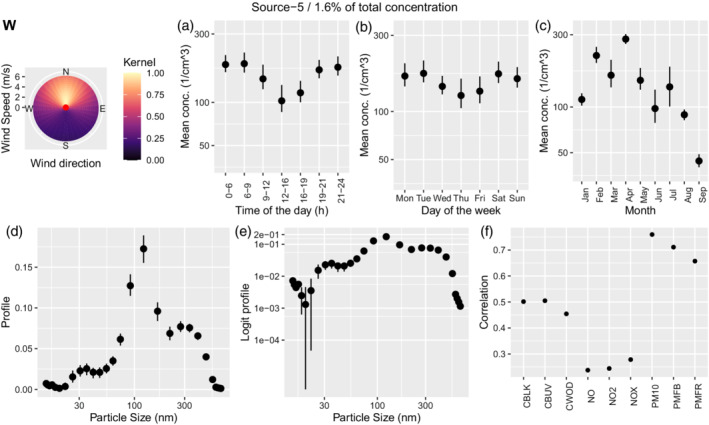
Characterization of source 5 which was identified as secondary aerosols A. We show the corresponding wind kernel (W), mean variation across time of the day (a), weekday (b), month (c), profile (d), profile on the logit scale (e), and correlation of the time‐series of concentration with externally measured air pollutants (f).

**FIGURE 8:**
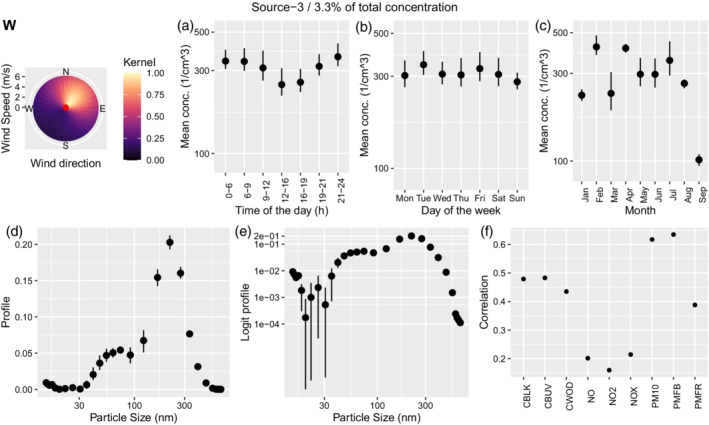
Characterization of source 3 which was identified as secondary aerosols B. We show the corresponding wind kernel (W), mean variation across time of the day (a), weekday (b), month (c), profile (d), profile on the logit scale (e), and correlation of the time‐series of concentration with externally measured air pollutants (f).

Two sources with multimodal profiles are presented in Figures 7 and 8 with 1.6% and 3.3% of the total concentration, respectively. Both show temporal patterns with low values during midday and elevated values during the night. Source 5, which we identify as secondary aerosols A, shows modes at around 30, 120 nm, and potentially 300 nm. Correlations with other pollutants are strongest for PM${}_{10}$, PMFB, and PMFR. Source 3, which we identify as secondary aerosols B, shows modes at around 60 nm and at 270 nm with a strong positive correlation with PM${}_{10}$ and PMFB too.

### 4.3 Additional sources

**FIGURE 9:**
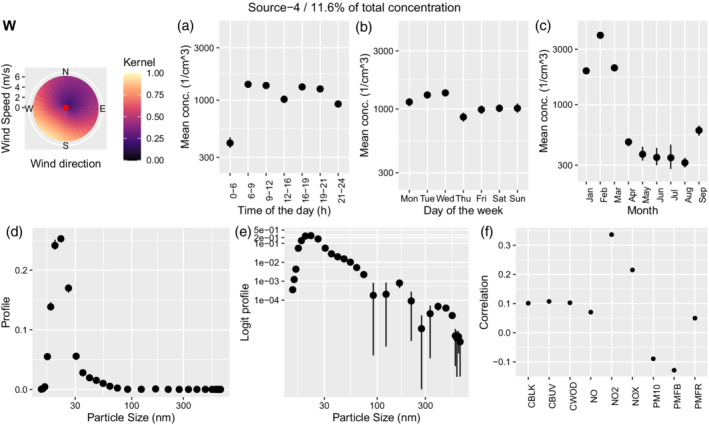
Characterization of source 4. We show the corresponding wind kernel (W), mean variation across time of the day (a), weekday (b), month (c), profile (d), profile on the logit scale (e), and correlation of the time‐series of concentration with externally measured air pollutants (f).

Besides the 6 sources found by Tremper et al. (2022) using traditional PMF, we find 3 additional sources. Source 4 (UKN winter), characterized in Figure 9, contributes 11.6% of the total concentration. The daily pattern is very similar to the source identified as airport but there is a strong seasonal pattern, with contributions in February 10‐fold those registered between May and August. The source profile shows a main peak around 20 nm and suggests two additional peaks around 170 and 400 nm. The source is positively correlated with NO${}_{2}$ and NO${}_{X}$, as can be seen in Figure 9f.

**FIGURE 10:**
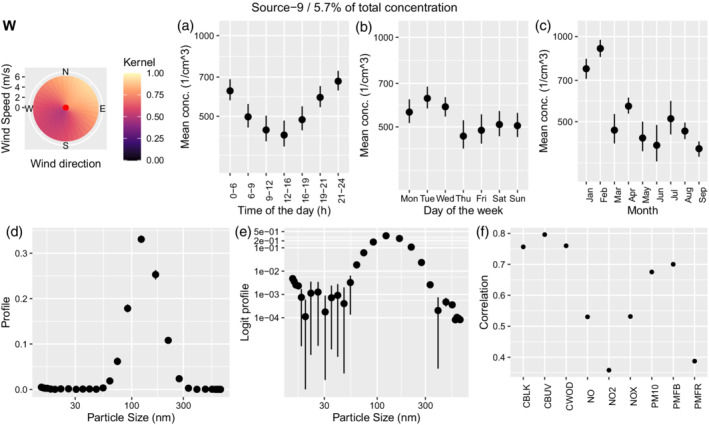
Characterization of source 9. We show the corresponding wind kernel (W), mean variation across time of the day (a), weekday (b), month (c), profile (d), profile on the logit scale (e), and correlation of the time‐series of concentration with externally measured air pollutants (f).

**FIGURE 11:**
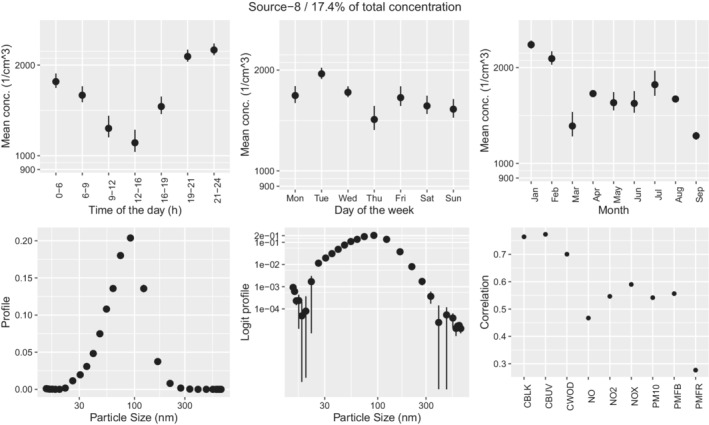
Characterization of source 8. We show the mean variation across time of the day (a), weekday (b), month (c), profile (d), profile on the logit scale (e), and correlation of the time‐series of concentration with externally measured air pollutants (f). Note that there is no wind kernel for source 8 because it was allocated as the Kth source by the model.

Sources 9 (UKN1) and 8 (UKN2) in Figures 10 and 11 show similar daily patterns to secondary aerosols with highest values during the night and lowest at midday. The profiles show a single peak above 100 nm for source 9 and just below 100 nm for source 8. The two sources are highly correlated and show similar correlations to other pollutants, hence could potentially be considered a single source.

## 5 DISCUSSION

In this article we presented a flexible statistical approach to apportion air pollution particles into their sources. Our Bayesian modeling framework is able to overcome some of the main limitations of factor analyses like PMF, which is the most widespread approach in the air pollution SA area. In particular, while the typical PMF is run several times with a different number of pre‐defined sources, in our data‐driven approach we specified a Dirichlet process prior on the source contribution, allowing us to estimate the number of components which contribute to the particle concentration without the need to fix their number. Additionally, we extended the DP framework to model temporal correlation across the mixing weights. We accounted for dependency in time through information on the wind (speed and direction) and by specifying a Gaussian kernel we were able to provide flexibility on the role of that covariate on the source allocation. At the same time, the flexibility of the framework makes it easily adjustable if: (i) it is not reasonable to model the temporal dependency through covariates, as an autoregressive structure can be included on the $\eta_{k,t}$ process; and (ii) additional covariates need to be included, which can be done via separate kernels. Finally, missing data in the concentration are naturally accounted for in the model through the posterior predictive distribution. However, missing values in the covariates need to be imputed. In our case a simple mean imputation was chosen because of the small number of missing values. In a sensitivity analysis we showed that results remain unchanged when time steps with missing covariates are discarded. Nevertheless, the framework could be easily extended to include a more complex imputation module as part of the model specification, which would allow for uncertainty on the imputed values to be fully taken into account and propagated.

Publications based on PMF report peaks of source profiles at higher accuracy than is possible in our model due to the aggregation of size bins (e.g., Tremper et al., 2022). It could be explored whether instead of deciding the aggregation of size bins in a purely data‐driven way aggregation can be avoided in the range between 20 and 40 nm where many peaks are expected. A further advantage of our model is that, in contrast to PMF, there is no need to specify measurement uncertainty for each size bin because the uncertainty is directly estimated in the model based on the residual variation.

We used the proposed approach to estimate air pollution sources using the concentration of particles of different sizes, as this metric is easy to collect and has been showed in previous papers to be able to discriminate sources. Comparing our results with the classical PMF, which was run on the same site on a slightly different temporal interval in Tremper et al. (2022), we were able to identify all 6 components previously found. In addition, our model distinguished 3 additional sources of which one was only found during winter and would not be expected to appear in the sampling campaign by Tremper et al. (2022) from July to October. The other two sources could represent secondary sources. Including compositional data in addition to the size distribution would aid identification of the responsible chemical processes and origin of the sources. We also showed that the wind is an important factor in informing the source allocation, as helping capture the temporal evolution of source profiles, and our analysis clearly showed that the kernel is noticeably different across different sources. Running the model without the wind kernel leads to an increased number of sources indicating that the wind information supports apportionment of particle sizes into a concise number of sources. This stresses the importance of allowing more flexibility in the source allocation.

Our model is robust to changes in the prior on α, the parameter controlling the behavior of the stick‐breaking process within the DPP. The number of sources found neither changes under a more informative prior that encourages accumulation into more sources, nor under a more vague prior that allows for accumulation into a single source, and settles in the order of 2.7 leading to a stick‐breaking procedure with a median break‐off distance at 22% and a probability of breaking off under 66% of 95%.

To conclude, we showed that the proposed method provides a flexible framework for SA, yet showing interpretable results. We believe that it is both, a promising alternative to the commonly used factor analysis based methods, and a complementary method to PMF, strengthening the confidence in the robustness of the found sources under different models. Further work will focus on the evaluation of source‐specific health effects associated with different particle size distribution in the air. Additionally, to enhance reproducibility and to allow non‐expert users to easily apply the proposed Bayesian approach to their own data, we aim at developing an user‐friendly R package, which will integrate MCMC programming for SA with a user‐interface which is simple and approachable.

## CONFLICT OF INTEREST

The authors declare no potential conflict of interests.

## Supporting information


**Data S1**: Supplementary Material
