# Supplementary material for: A dependent Bayesian Dirichlet process model for source apportionment of particle number size distribution
Source: Environmetrics. 2022 Sep 22;34(1):e2763. doi: 10.1002/env.2763 (PMC10077992; doi:10.1002/env.2763)
Supplement: Supplementary file 1 — Data S1: Supplementary Material [file ENV-34-0-s001.pdf]

Supplementary Material: A dependent Bayesian Dirichlet Process model for source apportionment of particle number size distribution

Oliver Baerenbold Melanie Meis Israel Martínez-Hernández Carolina Euán
Wesley S. Burr Anja Tremper Gary Fuller Monica Pirani
Marta Blangiardo

6/15/2022

1 Additional Figures

In this section, we include multiple figures that support the preprocessing steps and some data analysis conclusions.

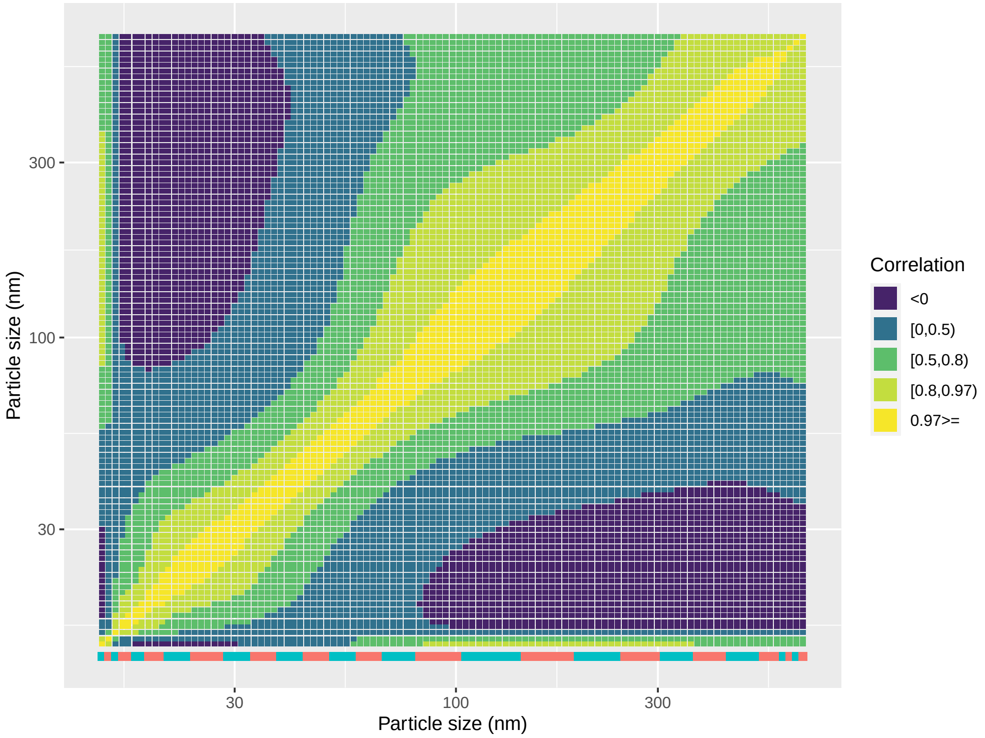

Figure 1: Correlation matrix between data in original size bins. Resulting aggregated bins are indicated at the bottom by colored segments.

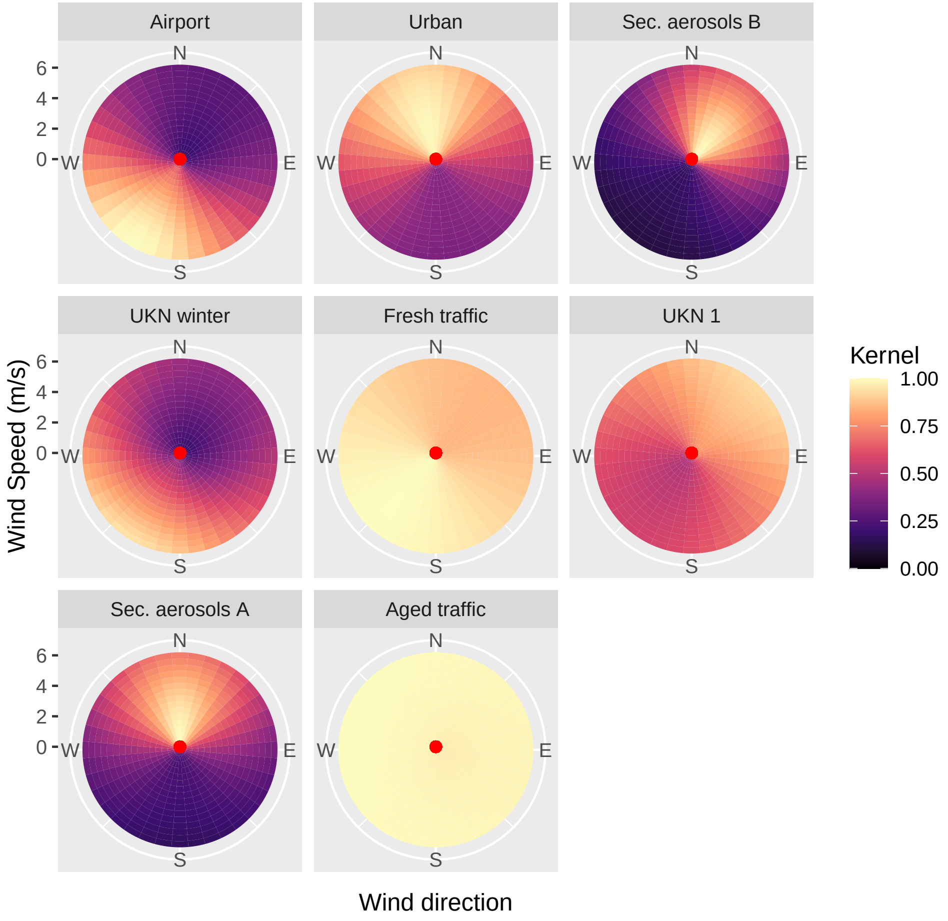

Figure 2: Wind kernels per identified source. Note that due to the finite approximation of the DPP, the model estimates only wind kernels for the first 8 sources.

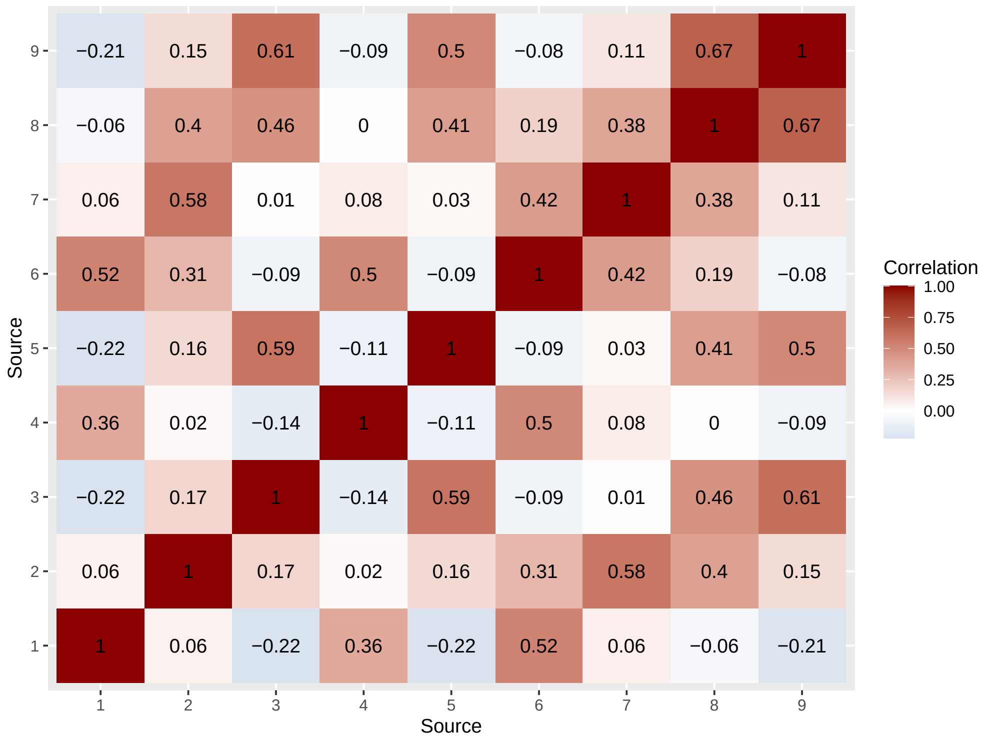

Figure 3: Correlation matrix between identified sources over time. Significant positive correlations are observed between sources 1 and 6; 2 and 7; 3, 5, and 9; and 9, and 8.

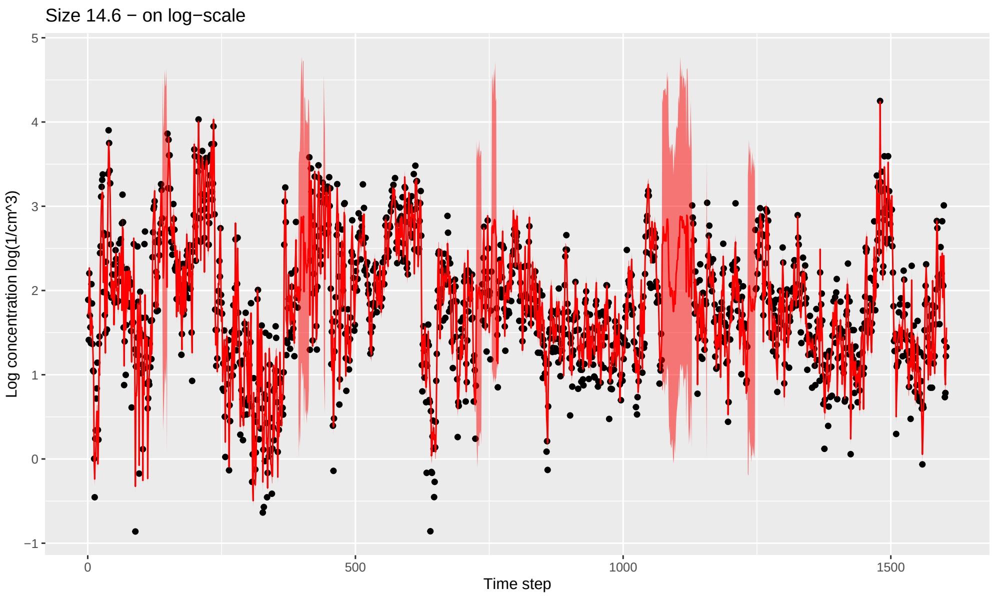

Figure 4: Predicted log concentrations (red line) for small size particles of 14 nm compared to observed values (black dots).

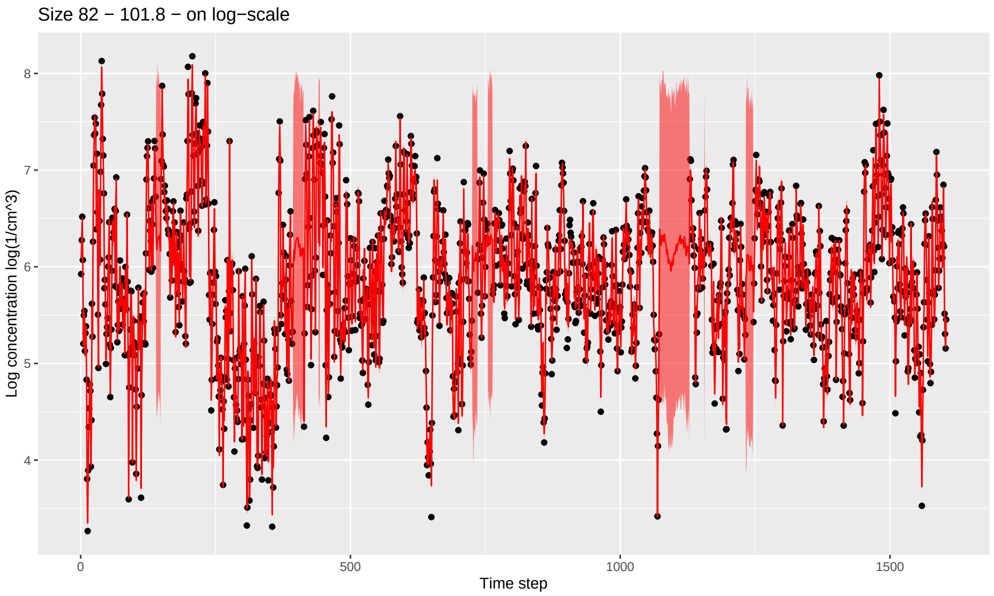

Figure 5: Predicted log concentrations (red line) for medium size particles of 100 nm compared to observed values (black dots).

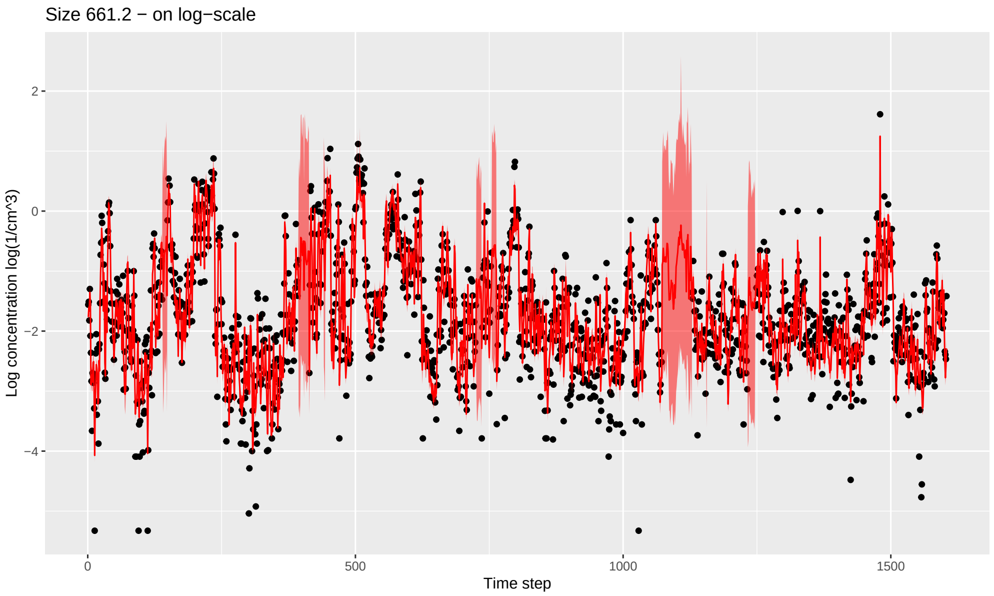

Figure 6: Predicted log concentrations (red line) for large size particles of 660 nm compared to observed values (black dots).

2 Comparison of different runs

2.1 Sensitivity to the prior

The α parameter can be understood as an inverse variance and broadly speaking, it controls the number of components. Being a key parameter in the model, we first consider a minimally-informative Gamma prior ($\alpha \sim \Gamma(1, 1)$), but perform sensitivity analysis changing it to a more ($\alpha \sim \Gamma(1, 10)$) and a less informative prior ($\alpha \sim \Gamma(1, 0.1)$), which would give higher and lower probability to a smaller number of components.

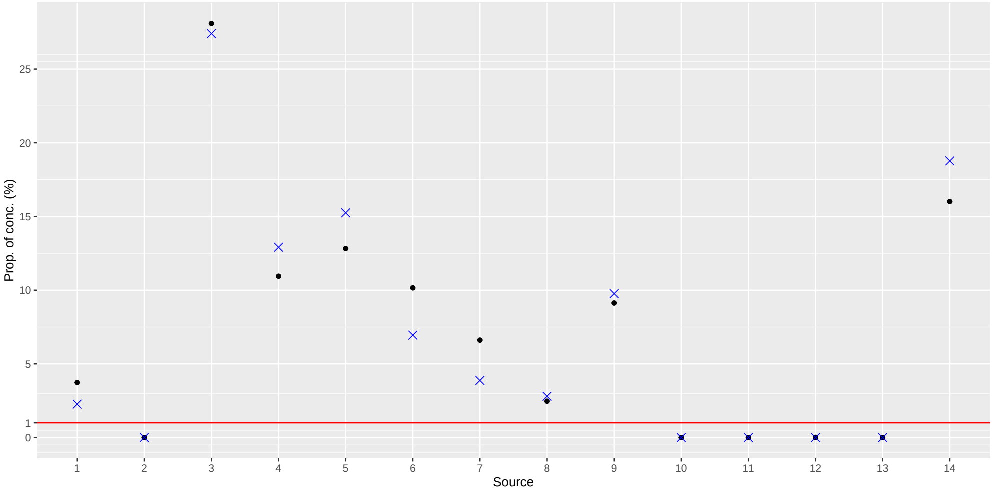

Figure 7: Mean proportion of concentrations per each source using a $\Gamma(1, 10)$ prior (black dots) and a $\Gamma(1, 0.1)$ prior (blue times) for the concentration parameter α. By considering only the sources with more than 1%, we would select 9 sources with both priors.

2.2 Inferences with a small and larger sample.

We ran two separate inferences, one with 120,000 iterations and 60,000 burn-in with 1 in 60 thinning (final sample used in the main paper), and one with 60,000 iterations and 30,000 burn-in with 1 in 30 thinning. Inferences were compared by whether they identified both the same sources and the same total number of sources. Relabelling was applied based on sources profile. The smaller sample will identify seven sources if we consider the 1% criteria. It could be possible that the smaller sample was not sufficient to identify all sources better. Given the sensitivity analysis results and the larger sample results, we decided to keep nine sources in the main paper.

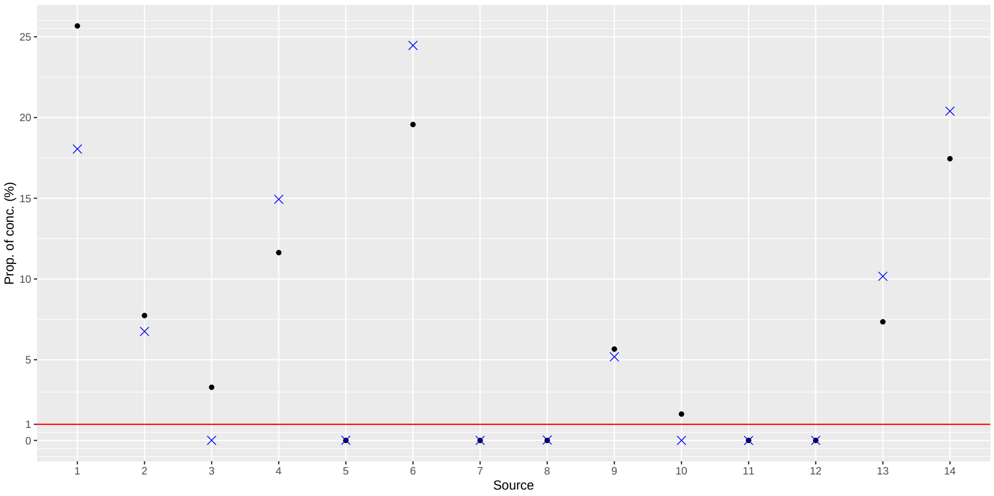

Figure 8: Mean proportion of concentrations per each source using a $\Gamma(1, 1)$ prior for the concentration parameter α. Blue times: 60,000 iterations and 30,000 burn-in with 1 in 30 thinning. Black dots: 120,000 iterations and 60,000 burn-in with 1 in 60 thinning.

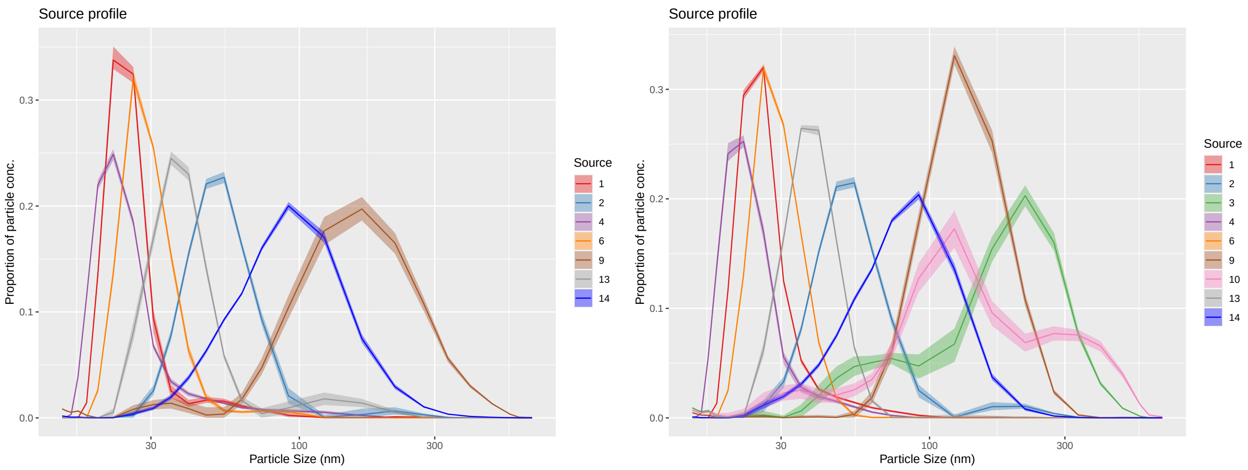

Figure 9: Particle size distribution of the identified sources. Left: 60,000 iterations and 30,000 burn-in with 1 in 30 thinning. Right: 120,000 iterations and 60,000 burn-in with 1 in 60 thinning.

3 Convergence in the MCMC chain

3.1 Load the Data

The full output of the Nimble run as summarized and discussed in the paper is available on Zenodo.

```
load("nimble_noar_r1_c14-_T_1604_comps_1-28_2022-02-26.RData")
load("data/data_base.RDAta")
```

We consider a single chain at this point to not have to implement label switching but split the chain to have access to more advanced diagnostics.

```
S_split <- ggs(samples$samples, splitting = TRUE)
```

Here we present some selected diagnostic plots since the number of parameters is considerably large. If any parameter set is not in this supplementary material, this can be investigated using similar codes and materials within the Git repository here.

3.2 Mean proportion of total concentration, $\bar{s}_k = 1/T \sum_t s_{k,t}$. Code notation: $s_{k,t}$ =p[k,t].

We consider as non-empty sources those with mean proportion above 1%. Selected indexes are 1, 2, 3, 4, 6, 9, 10, 13, and 14. Note that we re-label the sources in the main paper for better visualizations.

3.2.1 Trace plots

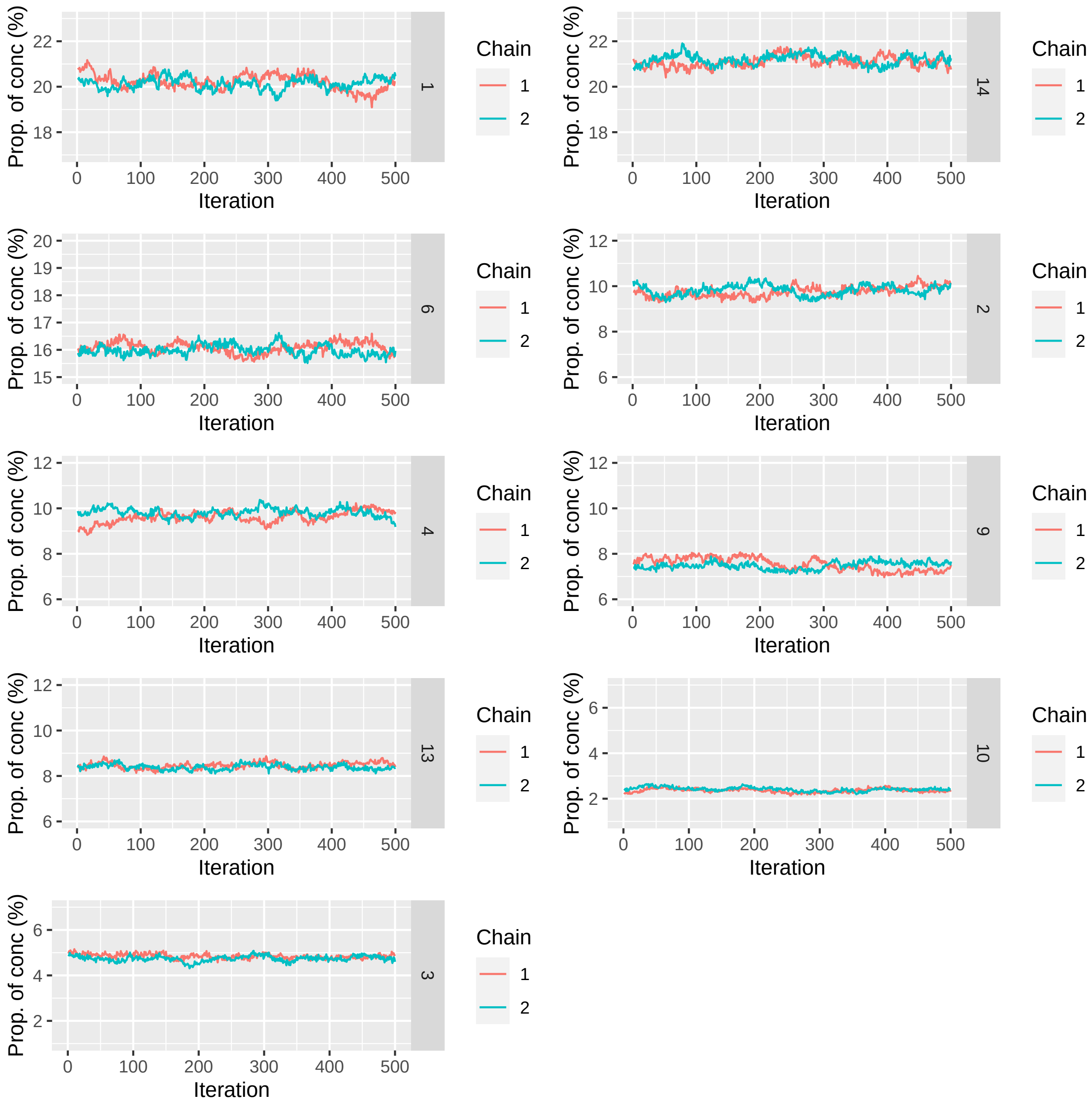

(empty clusters)

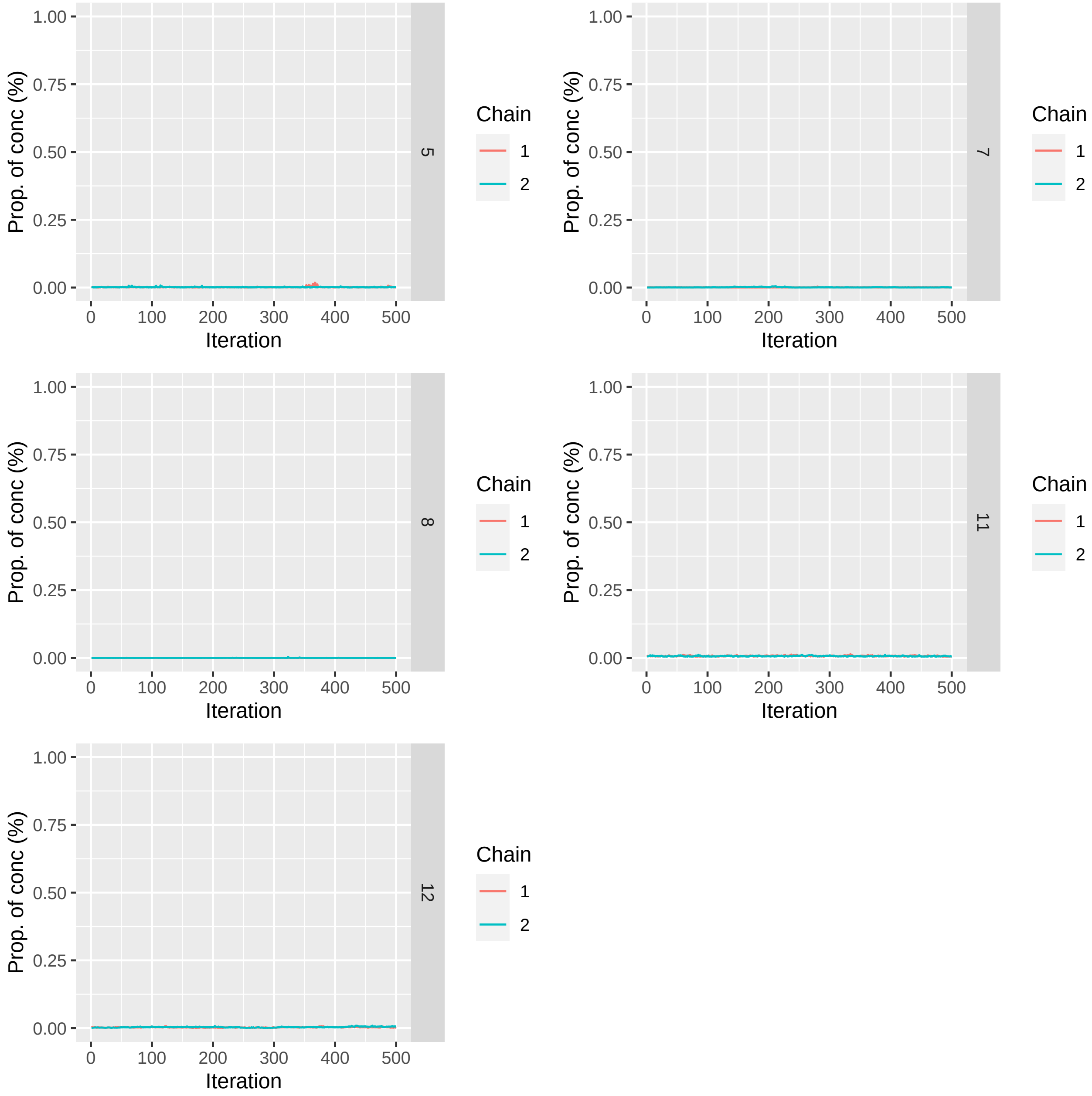

3.3 Source profile densities, $\lambda_{p,k}$, for $p = 1, \ldots, 28$ and $k = 1, \ldots, 9$. Code notation: $\lambda_{p,k}$ =mu.theta[p,k].

3.3.1 Trace plots for $p = 22.1, 62.7, 166.1, 552.5$ and $k = 1, 2, \ldots, 9$.

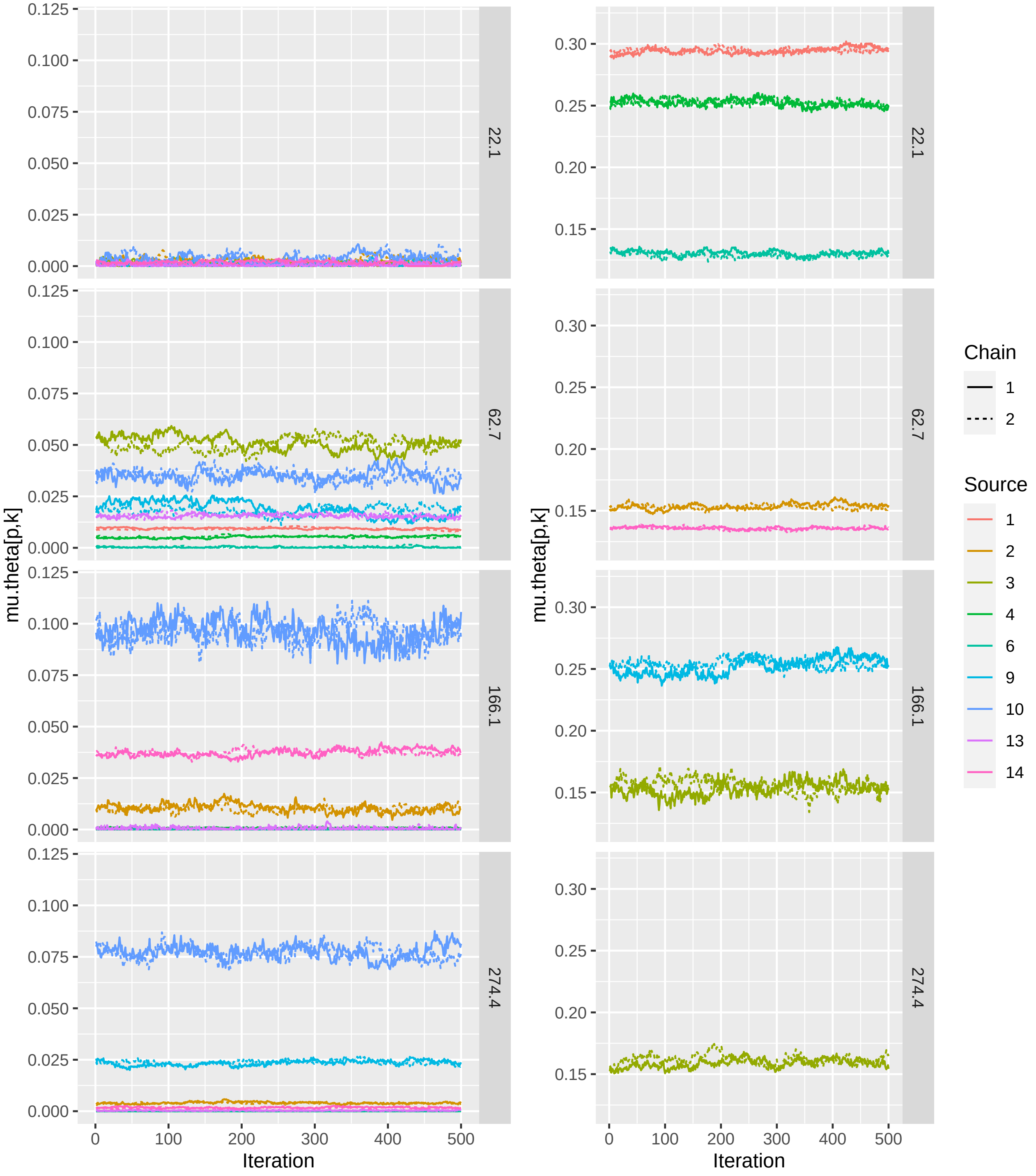

3.3.2 Rhat for source profile densities, $\lambda_{p,k}$.

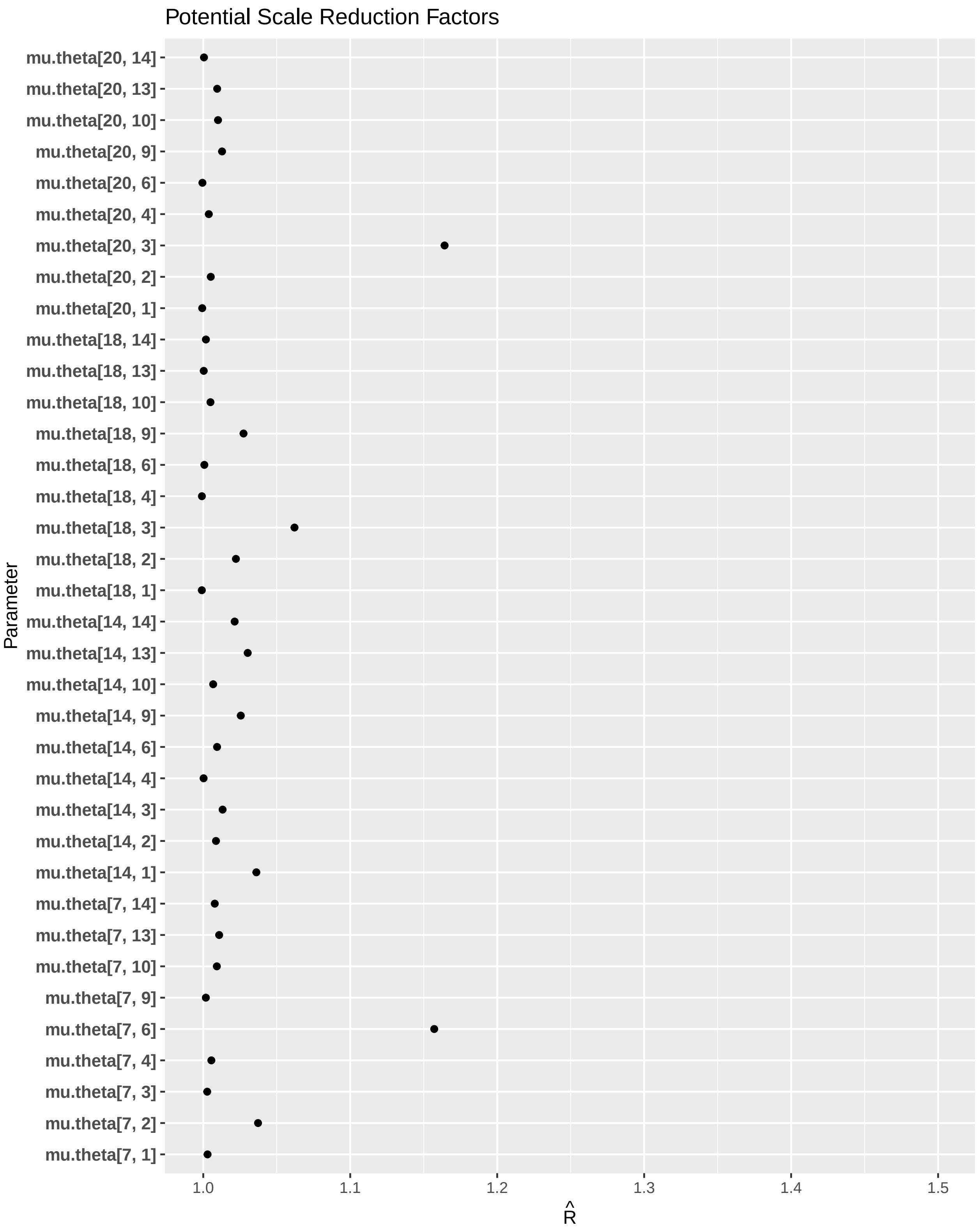

3.4 Precision (or concentration) parameter, α. Code notation: α=prec[1].

3.4.1 Trace plots for α

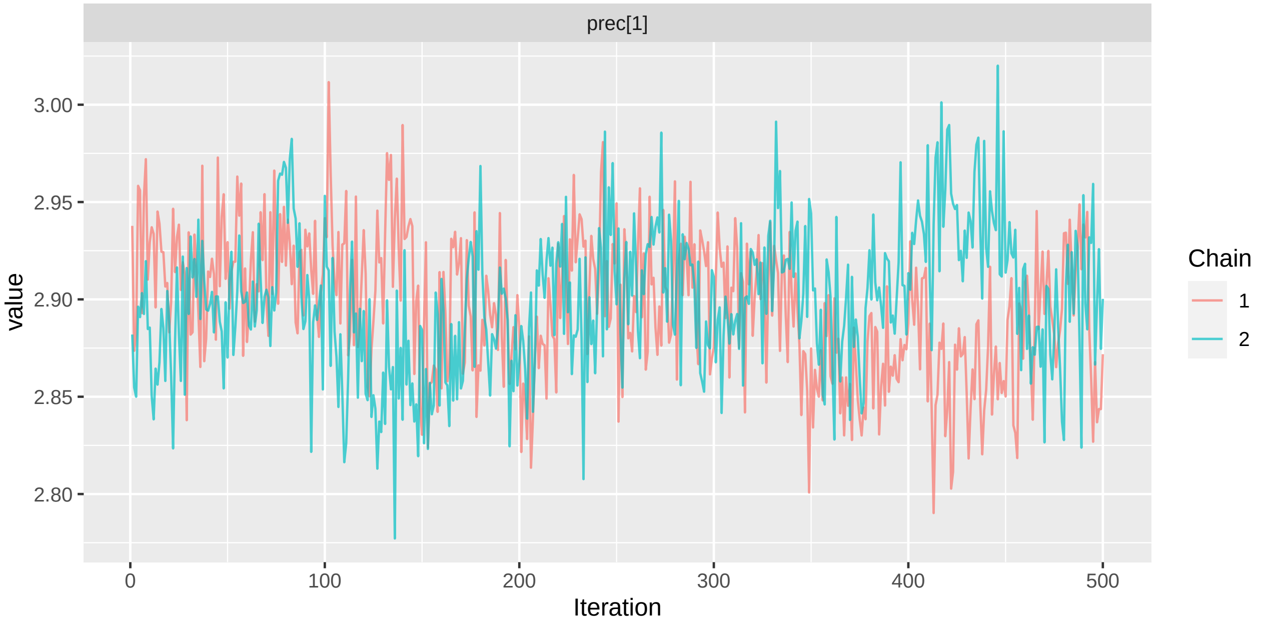

For the α parameter, Rhat = 1.
